# Plumbagin ameliorates LPS‐induced acute lung injury by regulating PI3K/AKT/mTOR and Keap1‐Nrf2/HO‐1 signalling pathways

**DOI:** 10.1111/jcmm.18386

**Published:** 2024-07-11

**Authors:** Zhengjia Liu, Jiahui Wei, Hongbin Sun, Lei Xu

**Affiliations:** ^1^ Department of Thoracic Surgery China‐Japan Union Hospital of Jilin University Changchun China; ^2^ Department of Respiratory China‐Japan Union Hospital of Jilin University Changchun China

**Keywords:** ALI, Keap1‐Nrf2/HO‐1 signalling, PI3K/AKT/mTOR signalling, plumbagin

## Abstract

Acute lung injury (ALI) is a major pathophysiological problem characterized by severe inflammation, resulting in high morbidity and mortality. Plumbagin (PL), a major bioactive constituent extracted from the traditional Chinese herb *Plumbago zeylanica*, has been shown to possess anti‐inflammatory and antioxidant pharmacological activities. However, its protective effect on ALI has not been extensively studied. The objective of this study was to investigate the protective effect of PL against ALI induced by LPS and to elucidate its possible mechanisms both in vivo and in vitro. PL treatment significantly inhibited pathological injury, MPO activity, and the wet/dry ratio in lung tissues, and decreased the levels of inflammatory cells and inflammatory cytokines TNF‐α, IL‐1β, IL‐6 in BALF induced by LPS. In addition, PL inhibited the activation of the PI3K/AKT/mTOR signalling pathway, increased the activity of antioxidant enzymes CAT, SOD, GSH and activated the Keap1/Nrf2/HO‐1 signalling pathway during ALI induced by LPS. To further assess the association between the inhibitory effects of PL on ALI and the PI3K/AKT/mTOR and Keap1/Nrf2/HO‐1 signalling, we pretreated RAW264.7 cells with 740Y‐P and ML385. The results showed that the activation of PI3K/AKT/mTOR signalling reversed the protective effect of PL on inflammatory response induced by LPS. Moreover, the inhibitory effects of PL on the production of inflammatory cytokines induced by LPS also inhibited by downregulating Keap1/Nrf2/HO‐1 signalling. In conclusion, the results indicate that the PL ameliorate LPS‐induced ALI by regulating the PI3K/AKT/mTOR and Keap1‐Nrf2/HO‐1 signalling, which may provide a novel therapeutic perspective for PL in inhibiting ALI.

## 1 INTRODUCTION

Acute lung injury (ALI) and its severe form, acute respiratory distress syndrome (ARDS), are respiratory distress conditions caused by numerous direct and indirect factors, resulting in progressive hypoxemia.^1^ ALI/ARDS are life‐threatening conditions, accounting for approximately 10% of ICU admissions.^2^ Despite significant progress in ALI pathophysiology and treatment, the morbidity rate remains high, with a mortality rate of approximately 50%, respectively.^3^, ^4^ Glucocorticoids are commonly used to treat ALI. However, their clinical is often limited due to the side effects such as drug resistance, metabolic disorders and osteoporosis.^5^, ^6^ Therefore, it is of great significance to develop safe and effective therapeutic drugs to treat ALI.

The phosphoinositide 3 kinase (PI3K)/AKT/mammalian target of rapamycin (mTOR) signalling pathway plays an important role in immunomodulation and inflammation, as well as in the cell metabolism, growth and proliferation.^7^ Recent studies have proved that PI3K/AKT/mTOR signalling is associated with a variety of diseases, including cancers,^8^ diabetic kidney disease^9^ and ALI.^10^ Evidences have showed that the levels of p‐PI3K, p‐AKT and p‐mTOR in lung tissues significantly decreased after ischemia/reperfusion (I/R) and the activation of PI3K/AKT/mTOR pathway significant inhibited the lung injury.^11^ Wen et al. demonstrated that *Tetrahydropalmatine* exerted an inhibitory effect on the I/R‐induced lung injury by inhibiting the activation of PI3K/AKT/mTOR signalling pathway.^12^ In addition, Chen et al. suggested that capsaicin ameliorated oxidative stress, inflammatory response and apoptosis by inhibiting the activation of PI3K/AKT/mTOR signalling pathways during LPS‐induced ALI.^13^ Similarly, another study with rats showed that compound 511 inhibited immunosuppression induced by opioids by regulating the balance of Th1/Th2 and the activation of the PI3K/AKT/mTOR signalling pathway.^14^

The activation of PI3K/AKT/mTOR signalling is cruical in inhibiting oxidative stress. Tissue damages caused by oxidative stress is a significant factor in promoting lung injury after LPS treatment.^15^ Oxidative stress is an imbalance between the oxidative and antioxidant systems, and contributes to inflammation by activating transcription factors such as NF‐E2‐related factor 2 (Nrf2).^16^ Nrf2 is suppressed by kelch‐like ECH‐associated protein 1 (Keap1) in the cytosol under physiological conditions. However, in the presence of redox imbalance, Nrf2 dissociates from Keap1 and binds antioxidant response elements in the nucleus, promoting the expression of detoxification genes.^17^, ^18^ Furthermore, the Keap1/Nrf2/HO‐1 axis produces heme oxygenase 1 (HO‐1), a downstream target gene of Nrf2, which involved in oxidative stress and inflammation, particularly in the case of ALI.^19^ Therefore, targeting the PI3K/AKT/mTOR or Keap1/Nrf2/HO‐1 signalling pathway may be an important target for the developing effective drugs against ALI.

Plumbagin (PL, 5‐hydroxy‐2‐methyl‐naphthalene‐1,4‐dione) is a major bioactive compound extracted from the roots of the medicinal plant *Plumbago zeylanica*, exhibits multiple pharmacological activities, including anticancer,^20^ anti‐bacterial,^21^ wound healing^22^ and anti‐inflammatory properties.^23^ Evidence has shown that PL attenuates rheumatoid arthritis (RA) by inhibiting the activation of the NF‐κB signalling pathway.^24^ Others have suggested that PL can inhibit lung fibroblast proliferation and differentiation by regulating AKT/mTOR signalling.^25^ Sun et al., recent indicated that inhibition of the activation of P13K/AKT/mTOR signalling plays an important role in the anticancer effects of PL in tongue squamous cell carcinoma (TSCC).^26^ Additionally, mounting evidence suggested that PL also possessed anti‐oxidative properties by regulating Nrf2/HO‐1 signalling and decreasing the levels of inflammatory mediators.^27^ Studies have shown that PL can reduce ROS levels and increased glutathione (GSH), superoxide dismutase (SOD), glutathione S‐transferase (GST), catalase (CAT) and glutathione peroxidase (GPx) by activating of Nrf2.^28^, ^29^ Other study has also indicated that PL treatment significantly inhibits myocardial 1/R injury by activating Nrf2.^30^ However, it remains to be elucidated whether PL has an inhibitory effect on ALI, and whether its effect is related to the PI3K/AKT/mTOR and Keap1/Nrf2/HO‐1 signalling pathways.

Therefore, the aim of this study was to investigate the protective effects of PL on LPS‐induced ALI and to explore the relevant mechanism underlying PL's anti‐inflammatory properties during ALI.

## 2 MATERIALS AND METHODS

### 2.1 Animals

The specified pathogen‐free (SPF) C57BL/6J male mice (aged 6–8 weeks and weighing 18 ± 2 g) were purchased from Liaoning Changsheng Biotechnology Co., Ltd. (Benxi, China). All animals were housed in SPF conditions on a 12‐h light–dark cycle and had access to mineral water ad libitum. This study had been approved by the institutional Animal Care and Use Committee (IACUC) of Jilin University (Changchun, China).

### 2.2 Reagents and materials

LPS (L2880) and Plumbagin (PL, C_11_H_8_O_3_) were purchased from Sigma‐Aldrich (St. Louis, MO). 740Y‐P (purity ≥98.85%) and ML385 (purity ≥99.96%) were purchased from MedChemExpress (Shanghai, China). Mouse TNF‐α, IL‐6, and IL‐1β enzyme‐linked immunosorbent assay (ELISA) kits were purchased from Biolegend (San Diego, CA, USA). GSH, MPO, MDA and CAT determination kits were purchased from the Jiancheng Bioengineering Institute of Nanjing (Jiangsu, China). Antibodies against p‐PI3K, PI3K, p‐AKT, AKT, p‐mTOR, mTOR, Keap1, Nrf2, HO‐1 and GAPDH were purchased from Cell Signalling (Boston, MA, USA).

### 2.3 Animals model establishment and treatment

A mouse model of ALI induced by LPS was established as previously described.^31^ Briefly, after 1 week of acclimation, 40 mice were randomly divided into five groups, including control, LPS (10 mg/kg) and PL (5, 10 and 20 mg/kg) + LPS groups. The mice were intraperitoneally injected with PL (5, 10 and 20 mg/kg) for two consecutive days. At 1 h after the last PL administration, the mice were administered intratracheally with 10 mg/kg of LPS (dissolved in 50 μL PBS) to induce ALI. Control mice were given an equivalent amount of PBS. After 24 h of LPS infusion, the bronchoalveolar lavage fluid (BALF) was collected and the lung tissues were excised for histopathological examination or stored at −80°C for further analysis.

### 2.4 BALF collection and analysis

Mice were euthanized at 24 h post‐LPS stimulation, and BALF was collected by flushing the tracheal cannula with 0.5 mL of normal saline for four times. The samples were then centrifuged at 300 ***g*** for 10 min at 4°C for cell counts, and the supernatant was collected for protein and inflammatory cytokines detection.

### 2.5 Protein leakage in BALF

The protein levels in the BALF supernatant were quantified using the BCA protein assay kit (Thermo Fisher, Shanghai, China) according to the manufacturer's instructions.

### 2.6 Lung wet/dry weight ratio

The lung wet/dry weight ratio was commonly used to evaluate the degree of pulmonary oedema. The right lungs of mice were weighed and dried in a constant temperature oven at 65°C for 72 h to calculate the wet/dry weight ratio of lung tissues.

### 2.7 Lung H&E staining and pathological score

The left lungs of the mice were fixed with 4% paraformaldehyde for 48 h and embedded in paraffin, cut into 5 μm sections and stained haematoxylin and eosin (H&E). Then, the stained sections were analysed using a fluorescence microscope (Nikon, Tokyo, Japan). In addition, the parameters of lung pathological scoring were referred to the previously described and were slightly modified.^32^ The parameters including neutrophils in the alveolar and interstitial space, hyaline membranes and alveolar septal thickening. Each variable was assigned a score of 0–3 points based on its severity.

### 2.8 Lung MPO, MDA, GSH, CAT and SOD activities detection

The lung tissues were dissected and homogenate by extraction buffer and activities of MPO, MDA, GSH, CAT and SOD were measured by commercial detection kits according to the manufacturer's instructions.

### 2.9 Cell culture and treated

RAW 264.7 cells were cultured in high‐glucose DMED supplemented with 10% fetal bovine serum (FBS) and 1% (v/v) penicillin/streptomycin (Beyotime Biotechnology, Shanghai, China) in a humidified 5% CO_2_ atmosphere at 37°C. Cells were cultured in six‐well plates until cells density reached 80% density. Then, they were treated with PL (1, 2 and 4 μM) for 1 h, followed by the addition of LPS (1 μg/mL) dissolved in medium to stimulate the cells for 24 h. To examine the effect of PI3K/AKT/mTOR and Keap1‐Nrf2/HO‐1 signalling pathways on the protective effect of PL on LPS‐induced ALI, the cells were treated with 30 μM 740Y‐P (PI3K/AKT activator) or 5 μM ML385 (Keap1‐Nrf2/HO‐1 inhibitor) for 1 h prior to PL (4 μM) treatment. Finally, the cell samples and cell suspension were collected.

### 2.10 Cell viability assay

RAW 264.7 cells were seeded in 96‐well plates at a density of 1 × 10^4^ cells per cell with 200 μL media. After 24 h of incubation, the cells were treated with 1 μg/mL LPS and various concentrations of PL for 48 h. Then, 20 μL of MTT (5 mg/mL) was added into each well to and incubated for an additional 4 h at 37°C. After removing the supernatant, 200 μL of DMSO per well was added to dissolve the purple formazan crystals. Finally, the absorbance at 570 nm was measured using a microplate reader (Rapoo, Finland).

### 2.11 ELISA assay

The levels of TNF‐α, IL‐6 and IL‐1β in cell‐free BALF and RAW 264.7 cells culture suspension were determined using ELISA kits by following the manufacturers' instructions.

### 2.12 Western blot

The lung tissues and cells were lysed by RIPA buffer with no added protease inhibitors (Beyotime Biotechnology, Shanghai, China). The protein content was detected by a BCA kit. Subsequently, the proteins were separated by SDS‐PAGE and transferred onto PVDF membranes (EMD Millipore). The membranes were blocked with 5% skimmed milk in TBST for 2 h and were incubated with the primary antibodies, including anti‐p‐PI3K, anti‐PI3K, anti‐p‐AKT, anti‐AKT, anti‐p‐mTOR, anti‐mTOR, anti‐Keap1, anti‐Nrf2, anti‐HO‐1 and anti‐GAPDH (CST, 1:1000) at 4°C for overnight. After that, the secondary antibody was added and incubated for 2 h. The target protein bands were formed with ECL reagents and quantified with ImageJ software.

### 2.13 Statistical analysis

All data were analysed using GraphPad Prism software. Data are presented as means ± SEM of at least three independent experiments. Only two group were analysed with Student's *t*‐test was used for two group comparisons, while one‐way analysis of variance followed by Tukey's test was used to more than two group comparisons. *p* < 0.05 was considered statistically significant.

## 3 RESULTS

### 3.1 The effect of PL on lung inflammatory response induced by LPS

**FIGURE 1:**
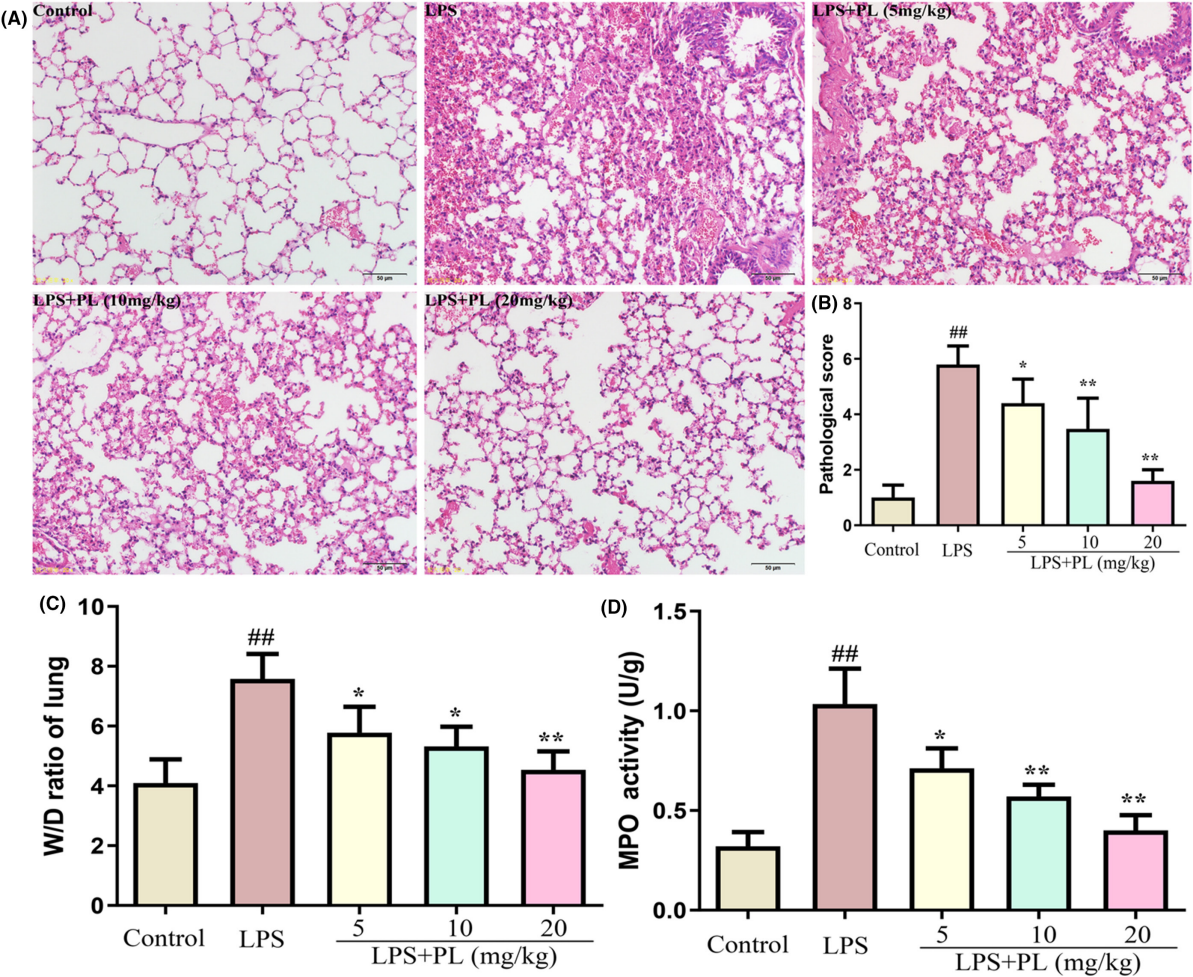
Effect of PL on lung inflammatory response induced by LPS. (A) Pathological changes of lung tissue (×100). (B) Pathological score of lung tissue. (C) Lung wet‐to‐dry weight ratio. (D) MPO activity in lung tissues. ^##^ *p* < 0.01 versus Control group; ^*^ *p* < 0.05, ^**^ *p* < 0.01 versus LPS group.

To examine the protective effect of PL on LPS‐induced ALI in mice, we assessed the pathological changes and pathological scores in lung tissues after LPS treatment. The results showed severe infiltration of inflammatory cell, and thickening of alveolar walls in lung tissue (Figure 1A). The lung pathological score was also markedly higher in the LPS treatment group compared with the control group (Figure 1B). In addition, the severity of lung oedema induced by LPS was confirmed by the increased levels of wet‐to‐weight ratio in lung tissues (Figure 1C). MPO activity, a classic biomarker, which represent the degree of neutrophil activation, were also obviously increased (Figure 1D). However, treatment with PL significantly inhibited the lung pathological damages and pathological score induced by LPS (Figure 1A,B). Furthermore, PL also reduced the lung wet/dry ratio and MPO activity during LPS‐induced ALI in mice (Figure 1C,D).

### 3.2 The effect of PL on inflammatory medium production in BALF induced by LPS

**FIGURE 2:**
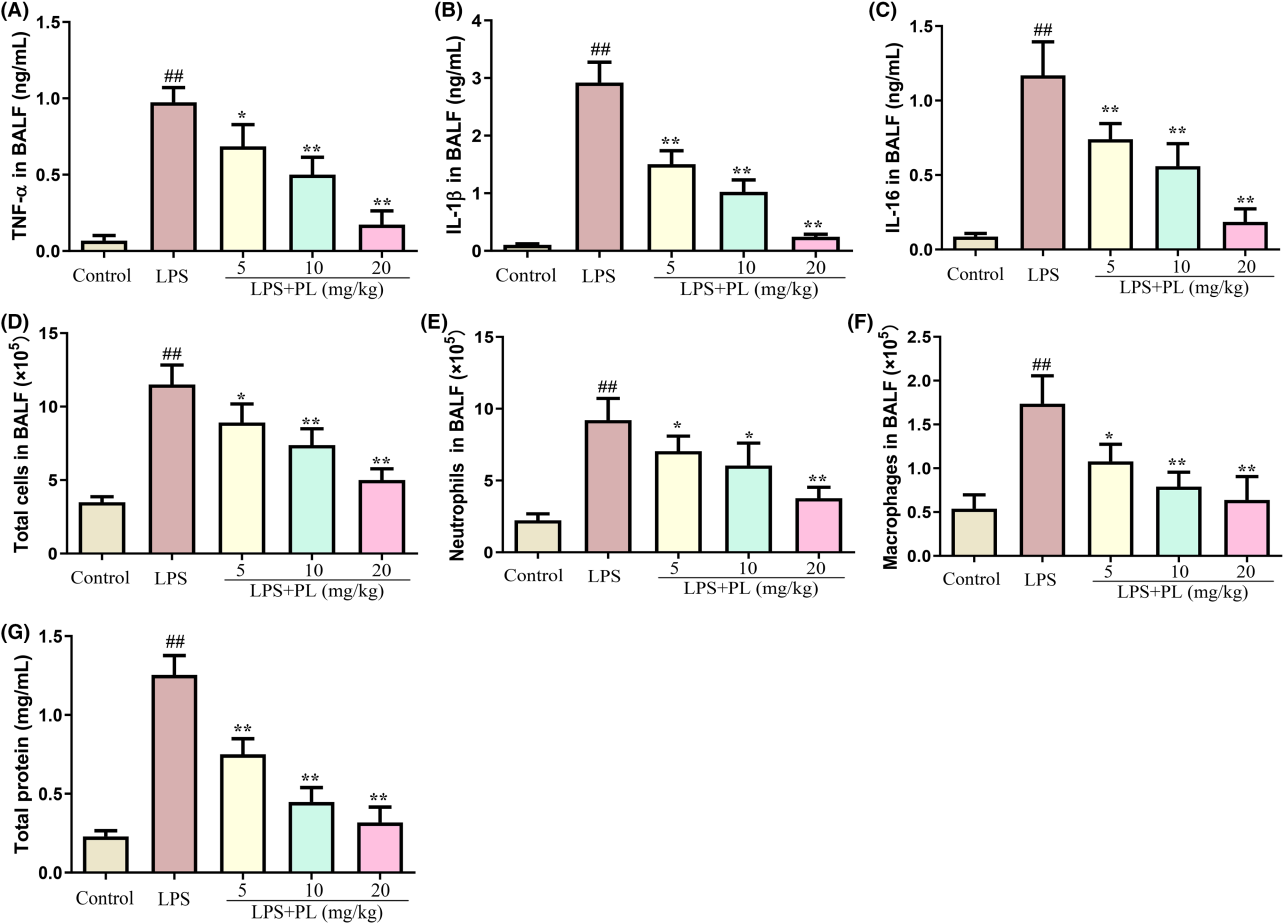
Effect of PL on inflammatory medium production in BALF induced by LPS. (A) The concentration of TNF‐α in BALF. (B) The concentration of IL‐1β in BALF. (C) The concentration of IL‐6 in BALF. (D) The level of total cells in BALF. (E) The level of neutrophils in BALF. (F) The level of macrophages in BALF. (G) The concentration of total protein in BALF. ^##^ *p* < 0.01 versus Control group; ^*^ *p* < 0.05, ^**^ *p* < 0.01 versus LPS group.

The levels of inflammatory cytokines and inflammatory cells in BALF were detected to assess the protective effect of PL on ALI. As shown in Figure 2A, the concentration of inflammatory cytokines TNF‐α, IL‐1β and IL‐6 was significantly increased in BALF after LPS stimulation but dose‐dependent reduced by PL treatment compared with the LPS treatment group mice. Moreover, the administration of LPS resulted in a significant increase in the levels of total cells, macrophages and neutrophils in BALF from mice. However, PL was able to inhibit this effect (Figure 2D–F). In addition, the concentration of total protein in BALF was used to assess the integrity of the lung barrier, and it was observed that PL treatment significantly reduced the concentration of total protein in BALF during ALI induced by LPS (Figure 2G). These results indicated that PL treatment effectively ameliorated LPS‐induced ALI in mice.

### 3.3 The effects of PL on PI3K/AKT/mTOR signalling during LPS‐induced ALI

**FIGURE 3:**
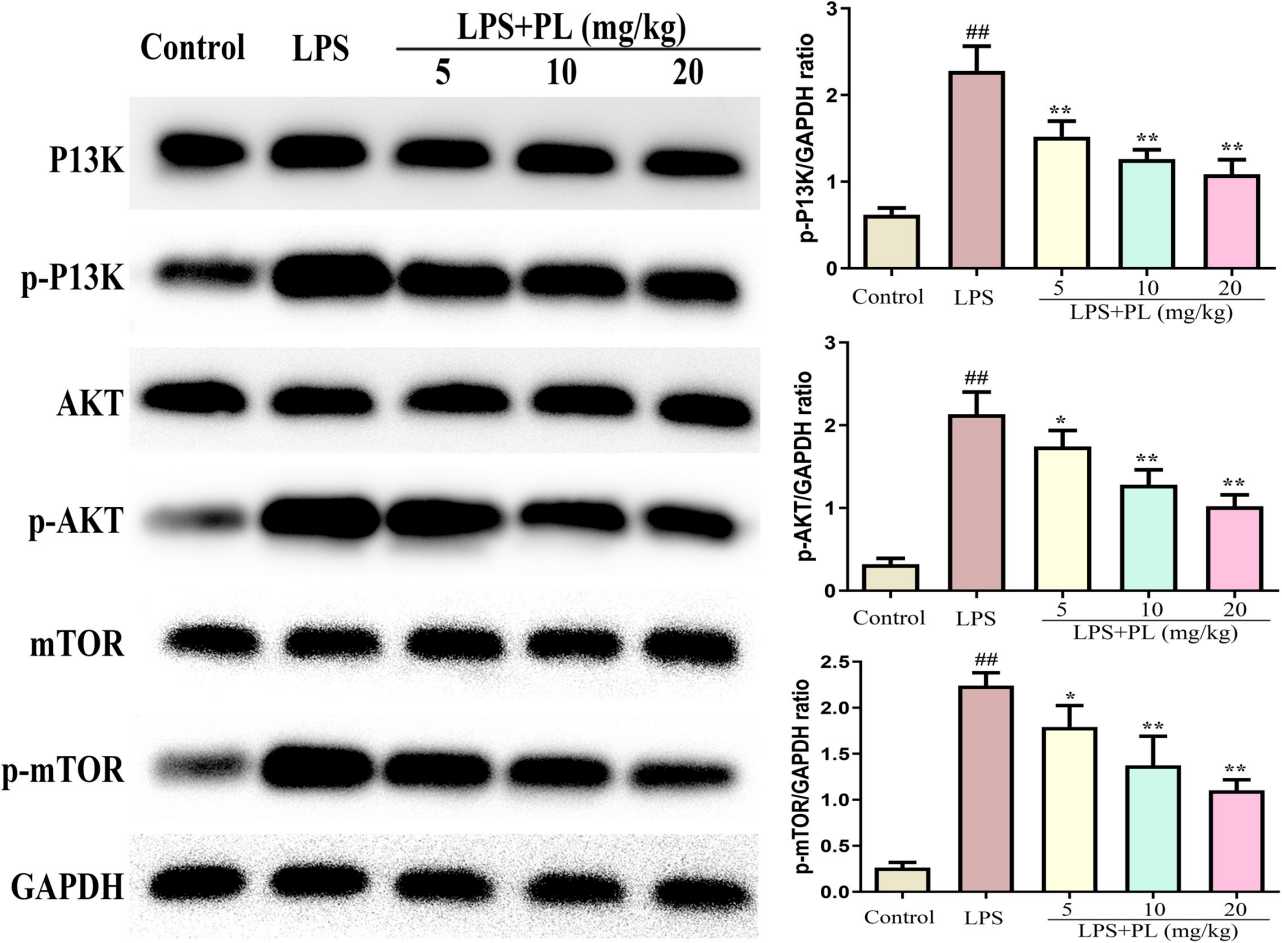
Effects of PL on PI3K/AKT/mTOR signalling during LPS‐induced ALI. The protein content of P13K, p‐P13K, AKT, p‐AKT, mTOR and p‐mTOR were detected by western blot, and GAPDH was used as internal reference protein. ^##^ *p* < 0.01 versus Control group; ^*^ *p* < 0.05, ^**^ *p* < 0.01 versus LPS group.

The PI3K/AKT/mTOR signalling plays an important in regulating the release of inflammatory cytokines and oxidative stress in ALI.^33^ Therefore, we detected the activation of the PI3K/AKT/mTOR signalling after treatment with PL during LPS‐induced ALI. As shown in Figure 3, the proteins expression of p‐PI3K, p‐AKT and p‐mTOR were significantly increased after LPS administration, and PL treatment inhibited the expression of these proteins compared with the LPS challenge group in a dose‐dependent manner. The results suggest that the PI3K/AKT/mTOR signalling pathway may have a vital impact on the anti‐ALI of PL.

### 3.4 The effect of PL on Keap1‐Nrf2/HO‐1 signalling during LPS‐induced ALI

**FIGURE 4:**
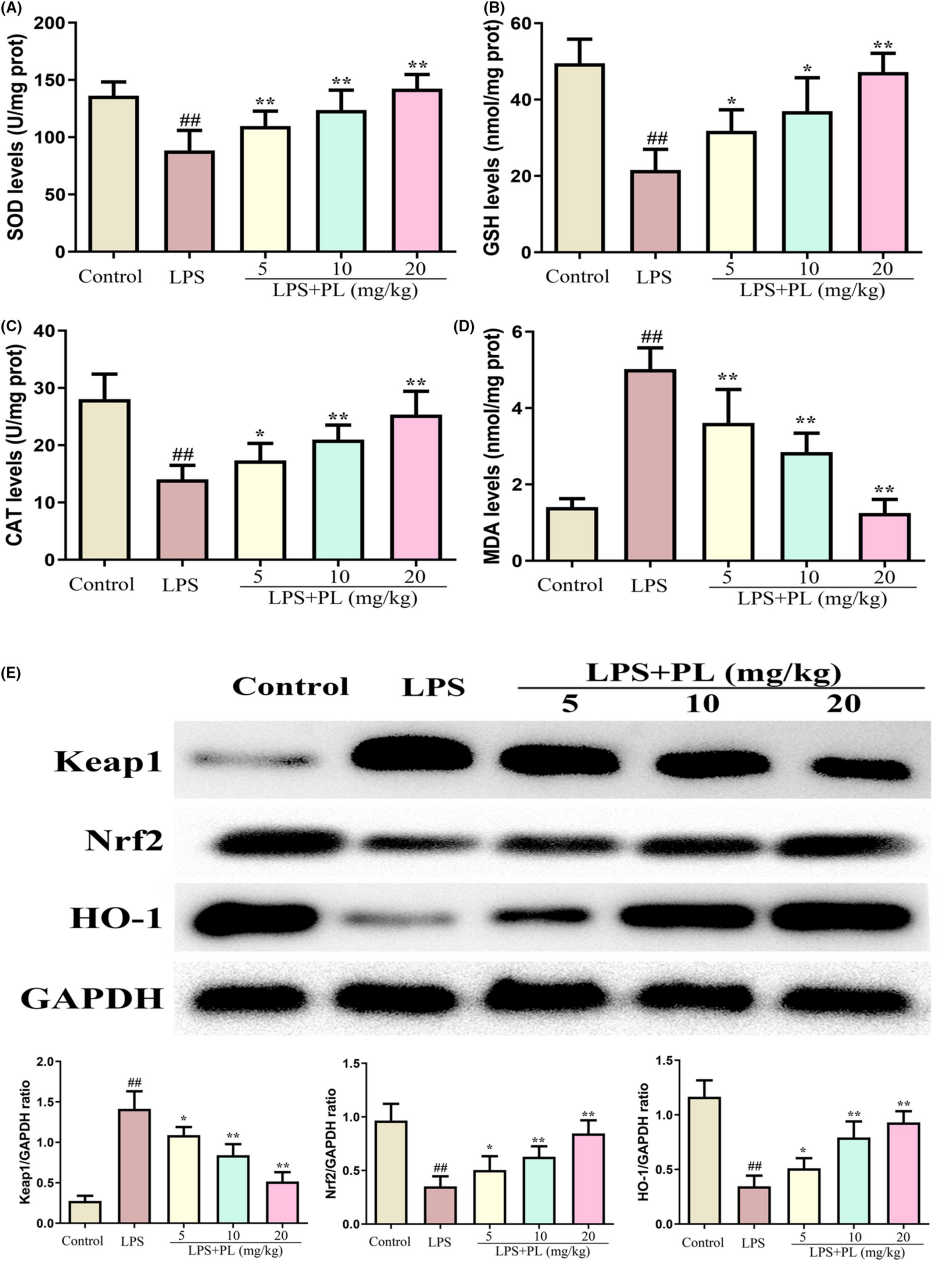
Effect of PL on Keap1‐Nrf2/HO‐1 signalling during LPS‐induced ALI. (A) SOD activity in lung tissues. (B) GSH activity in lung tissues. (C) CAT activity in lung tissues. (D) MDA activity in lung tissues. (E) The protein content of Keap1, Nrf2, and HO‐1 were detected by western blot, and GAPDH was used as internal reference protein. ^##^ *p* < 0.01 versus Control group; ^*^ *p* < 0.05, ^**^ *p* < 0.01 versus LPS group.

Oxidative stress can lead to ALI. To assess the role of PL on antioxidant status during LPS‐induced ALI, we detected the level of MDA, SOD, GSH and CAT in lung tissues. The results showed that LPS administration reduced the production of SOD, GSH, CAT and increased the production of MDA, while treatment of PL reversed those change induced by LPS (Figure 4A–D). In addition, the activation of antioxidant‐related signalling pathway of Keap1‐Nrf2/HO‐1 was detected by western blot. The results showed that PL treatment during LPS‐induced ALI led to a reduction in Keap1 protein expression and a significant increase in Nrf2 and HO‐1 protein expression in lung tissues (Figure 4E). These results suggested that the protective effect of PL on ALI may be associated with the activation of the Keap1‐Nrf2/HO‐1 signalling pathway.

### 3.5 The effect of PL on inflammatory response in the RAW264.7 cells induced by LPS

**FIGURE 5:**
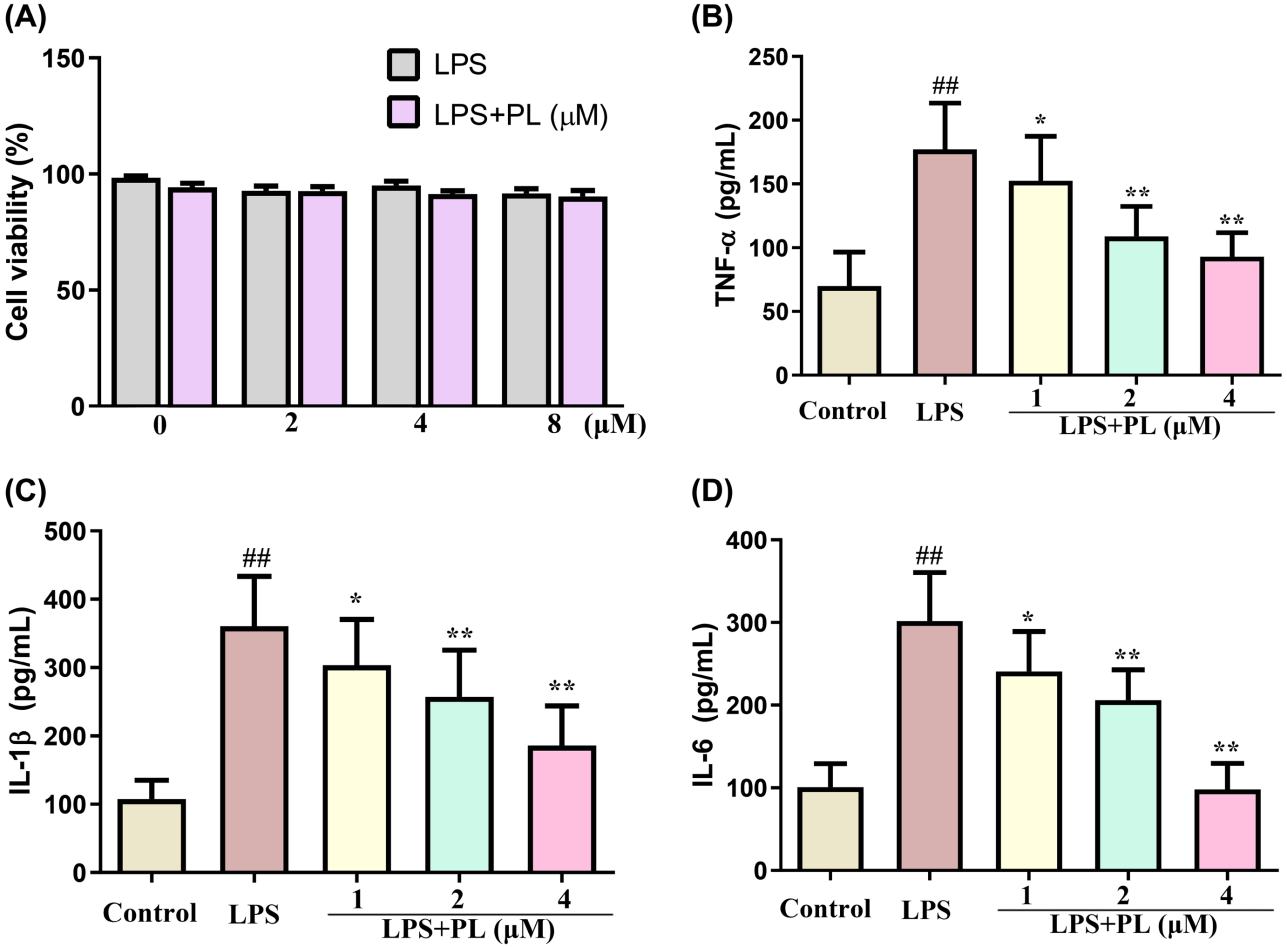
Effect of PL on inflammatory response in the RAW264.7 cells induced by LPS. (A) Viability of RAW264.7 cells after exposure to PL was detected by MTT. (B) The concentration of TNF‐α in cell‐cultured supernatant. (C) The concentration of IL‐1β in cell‐cultured supernatant. (D) The concentration of IL‐6 in cell‐cultured supernatant. ^##^ *p* < 0.01 versus Control group; ^*^ *p* < 0.05, ^**^ *p* < 0.01 versus LPS group.

The cytotoxic effect of PL on RAW 264.7 cells was assessed by MTT, and the results showed that PL is not cytotoxic to cells at concentrations below 8 μM for 24 h (Figure 5A). Therefore, the concentrations of 1, 2 and 4 μM PL were used in the subsequent experiments. To investigate the anti‐inflammatory role of PL in vitro, we measured the concentration of inflammatory cytokines TNF‐α, IL‐1β and IL‐6 in cell culture suspensions of RAW 264.7 cells from different treatment groups. The results showed that LPS treatment significantly increased the production of TNF‐α, IL‐1β and IL‐6 in cells compared with the control group cells; however, treatment with PL dose‐dependently diminished the stimulatory effects of LPS (Figure 5B–D). These results suggested that PL could inhibit the inflammatory response induced by LPS.

### 3.6 The effect of PL on PI3K/AKT/mTOR and Keap1‐Nrf2/HO‐1 signalling in the RAW264.7 cells induced by LPS

**FIGURE 6:**
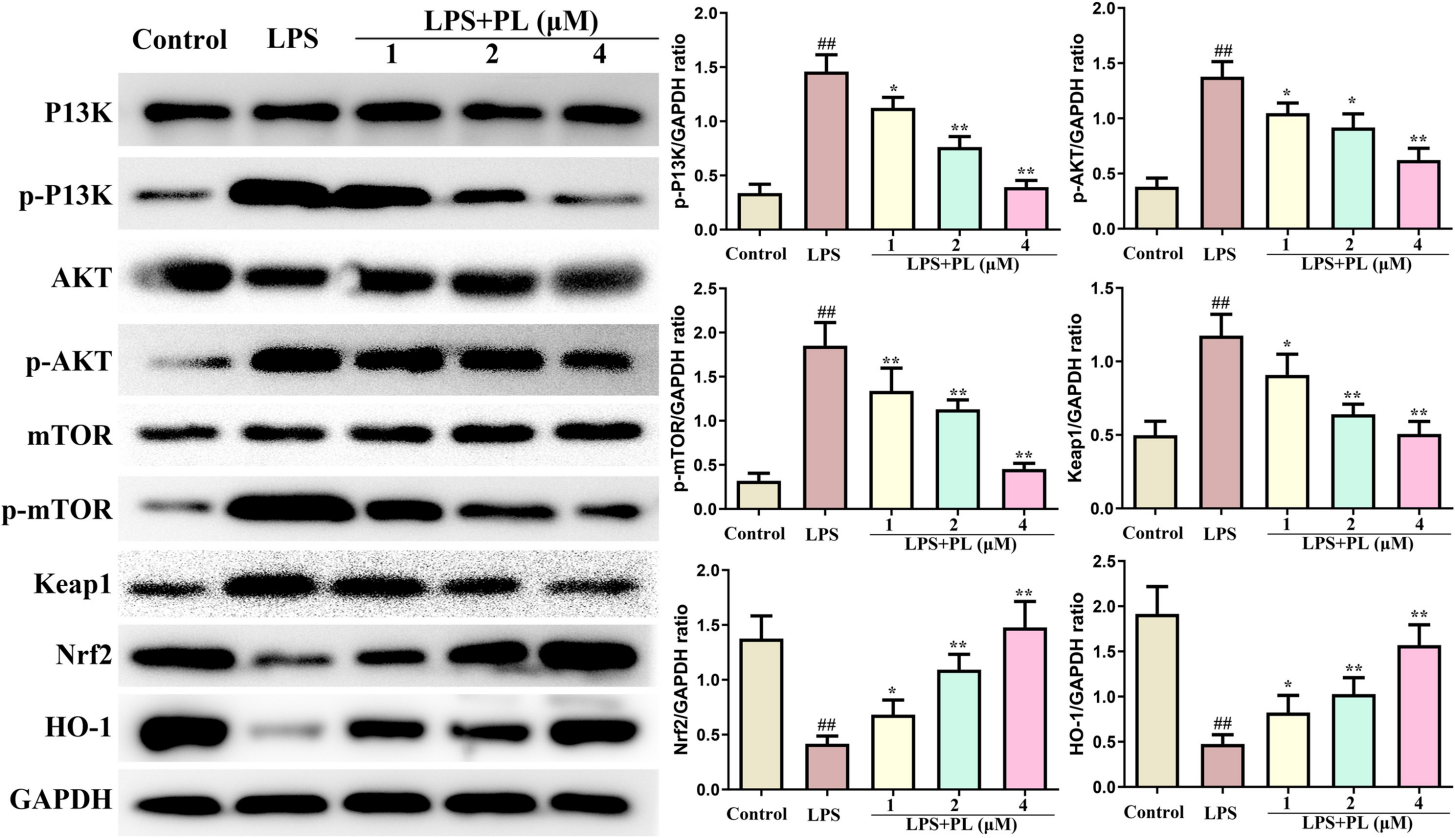
Effect of PL on PI3K/AKT/mTOR and Keap1‐Nrf2/HO‐1 signalling in the RAW264.7 cells induced by LPS. The protein content of P13K, p‐P13K, AKT, p‐AKT, mTOR, p‐mTOR, Keap1, Nrf2 and HO‐1 were detected by western blot, and GAPDH was used as internal reference protein. ^##^ *p* < 0.01 versus Control group; ^*^ *p* < 0.05, ^**^ *p* < 0.01 versus LPS group.

The changes of PI3K/AKT/mTOR and Keap1‐Nrf2/HO‐1 signalling pathway in RAW264.7 cells from different treatment groups were also detected by western blot. It is apparent from Figure 6 that LPS administration significantly increased the protein expression of p‐PI3K, p‐AKT and p‐mTOR, which was inhibited by PL. Furthermore, PL also reduced the expression of Keap1 and increased the expression of Nrf2 and HO‐1 induced by LPS. These results suggested that PL inhibited the activation of PI3K/AKT/mTOR and activated the Keap1‐Nrf2/HO‐1 signalling induced by LPS.

### 3.7 Activation of PI3K/AKT/mTOR signalling reversed the protective effect of PL on LPS‐induced inflammatory response in the RAW264.7 cells

**FIGURE 7:**
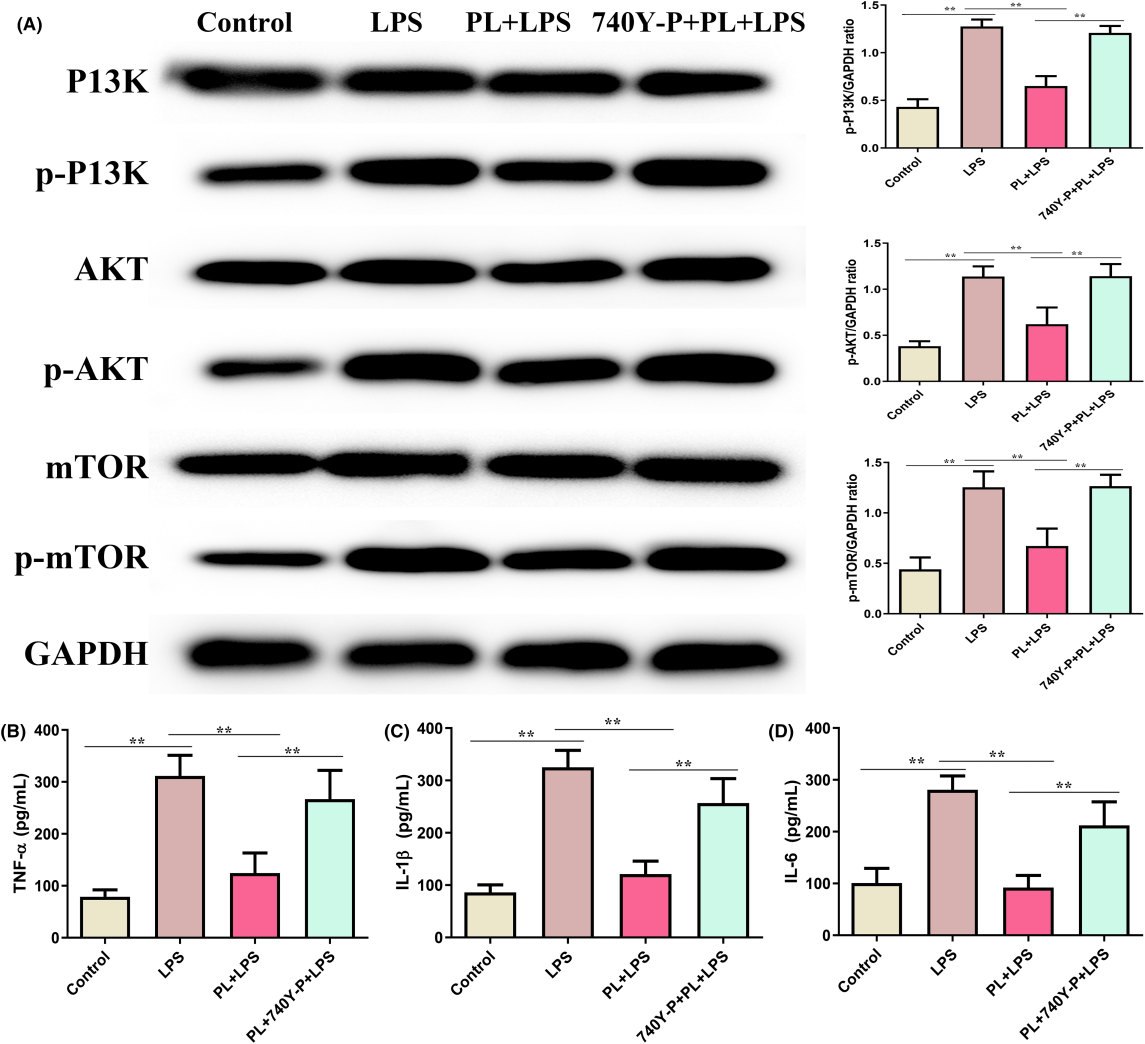
Activation of PI3K/AKT/mTOR signalling reversed the protective effect of PL on LPS‐induced inflammatory response in the RAW264.7 cells. (A) The protein content of P13K, p‐P13K, AKT, p‐AKT, mTOR and p‐mTOR were detected by western blot, and GAPDH was used as internal reference protein. (B) The concentration of TNF‐α in cell‐cultured supernatant. (C) The concentration of IL‐1β in cell‐cultured supernatant. (D) The concentration of IL‐6 in cell‐cultured supernatant. ^**^ *p* < 0.01.

To assess whether PL inhibits LPS‐induced ALI through regulating the PI3K/AKT/mTOR signalling, we detected the levels of inflammatory response induced by LPS after treatment with the PI3K/AKT agonist 740Y‐P. The results showed that the p‐PI3K, p‐AKT and p‐mTOR proteins levels, and inflammatory cytokines TNF‐α, IL‐1β and IL‐6 concentrations were increased in RAW264.7 cells treated with LPS (Figure 7A–D), which reversed the inhibition role of PL on LPS‐induced inflammatory response by treating with 740Y‐P (Figure 7A–D). These results suggested that the protective effect of PL on LPS‐induced ALI by inhibiting the activation of the PI3K/AKT/mTOR signalling pathway.

### 3.8 Inhibition of Keap1‐Nrf2/HO‐1 signalling reversed the protective effect of PL on LPS‐induced inflammatory response in the RAW264.7 cells

**FIGURE 8:**
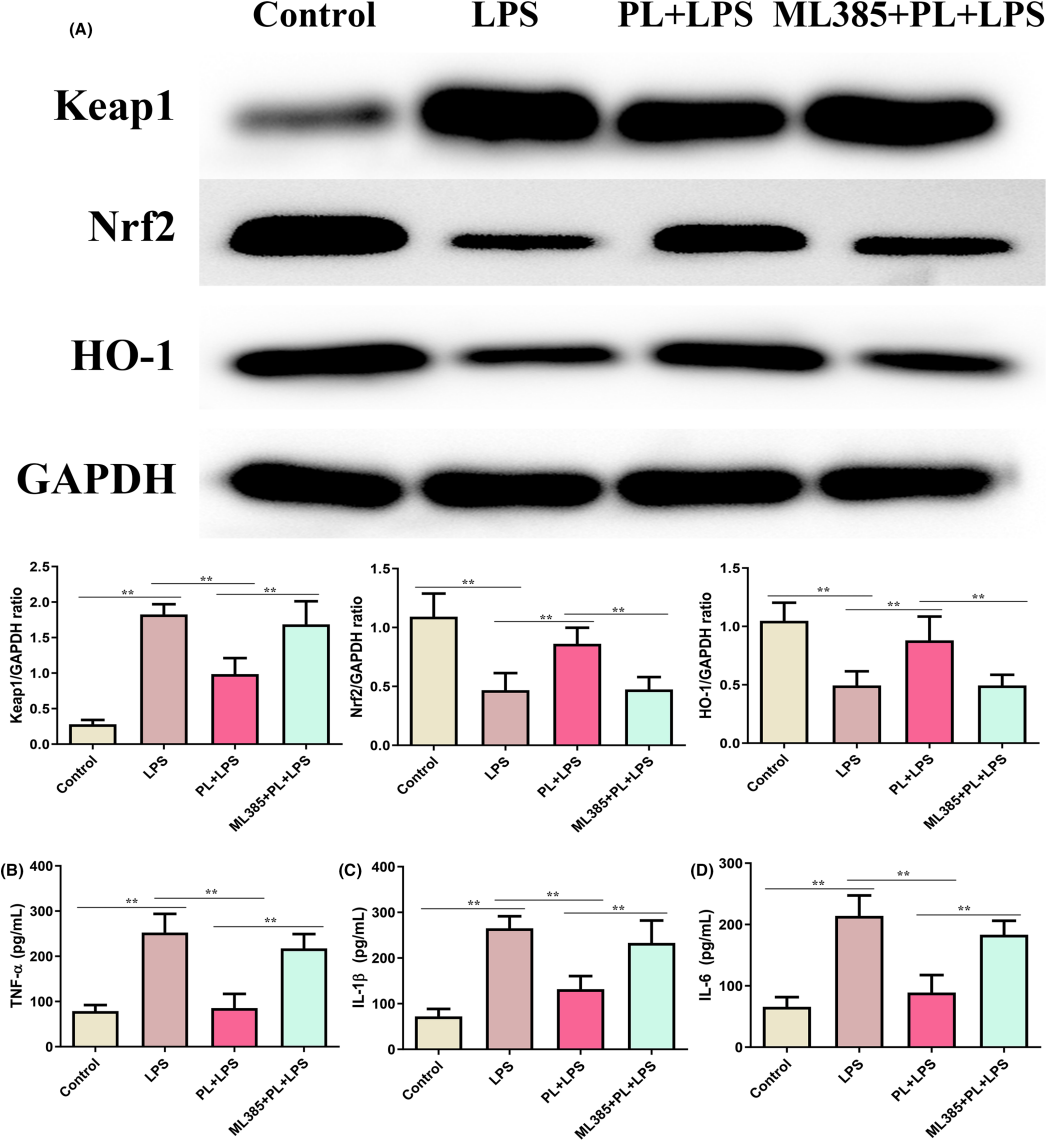
Inhibition of Keap1‐Nrf2/HO‐1 signalling reversed the protective effect of PL on LPS‐induced inflammatory response in the RAW264.7 cells. (A) The protein content of Keap1, Nrf2 and HO‐1 were detected by western blot, and GAPDH was used as internal reference protein. (B) The concentration of TNF‐α in cell‐cultured supernatant. (C) The concentration of IL‐1β in cell‐cultured supernatant. (D) The concentration of IL‐6 in cell‐cultured supernatant. ^**^ *p* < 0.01.

To better understand the anti‐inflammation function of PL, we further treated RAW264.7 cells with ML385 to inhibit the activation of Keap1‐Nrf2/HO‐1 signalling pathway. As shown in Figure 8A, PL treatment significantly increased the expression of Nrf2 and HO‐1, and administration of ML385 reduced the levels of these proteins from the PL + LPS treated group cells (Figure 8A). Moreover, the production of TNF‐α, IL‐1β and IL‐6 were notably increased after treatment with PL, ML385 and LPS, compared with the PL + LPS treatment group (Figure 8B–D). The results suggested that inhibiting the Keap1‐Nrf2/HO‐1 signalling weakened the anti‐inflammatory function of PL on LPS‐stimulated RAW264.7 cells.

## 4 DISCUSSION

ALI is a life‐threatening disease characterized by inflammation, epithelial and endothelial injury and coagulation disorders.^32^ Although significant strides have been made in understanding the pathophysiological mechanisms of ALI in recent decades, the search for specific drugs and effective therapies remains limited. Previous studies have provided evidence that the PI3K/AKT/mTOR and Keap1‐Nrf2/HO‐1 signalling pathways are involved in the development of ALI,^34^, ^35^ and traditional Chinese herbal medicine PL can exert its pharmacologic effect by regulating the PI3K/AKT/mTOR and Nrf2 signalling pathway.^30^, ^36^ Thus, we assessed the protective effects and mechanisms of PL on LPS‐induced ALI in vivo and in vitro. The results showed that treatment of PL significantly ameliorated LPS‐induced ALI in mice and inhibited the inflammatory response induced by LPS in RAW 264.7 cells. In terms of mechanism, the protective effect of PL on ALI was achieved by inhibiting the activation of PI3K/AKT/mTOR and activating Keap1‐Nrf2/HO‐1 signalling pathways.

To verify the protective effect of PL on ALI, we established an LPS‐induced ALI model in mice. We found that PL inhibited lung pathological injury and inflammatory cell infiltration. PL also reduced the pulmonary oedema, as showed by a decrease in lung wet/dry ratio. Moreover, total protein concentration as markers of pulmonary vascular permeability.^37^ We also found that PL reduced the concentration of total protein induced by LPS in BALF. In addition, PL treatment inhibited the overproduction of macrophages and infiltration of neutrophils, as well as the concentration of inflammatory cytokines TNF‐α, IL‐1β and IL‐6 in BALF induced by LPS.

The production of inflammatory cytokines was regulated by the translocation of NF‐κB, which plays a crucial role in triggering the inflammatory response in lung tissues.^18^ Studies have indicated that PI3K/AKT/mTOR signalling plays an important role in regulating the activation of inflammatory pathway NF‐κB, and the activation of the PI3K/AKT/mTOR signalling pathway is also associated with the pathological process of ALI.^12^ Evidence showed that activation of PI3K/AKT/mTOR signalling contributed to the development of ALI by inducing the maturation and antigen‐presenting ability of lung DCs.^38^ In addition, numerous studies have demonstrated that the inhibiting the PI3K/AKT/mTOR signalling can attenuate the ALI.^10^, ^13^ Lu et al. suggested that dexmedetomidine inhibited LPS‐induced ALI in rats by modulating the HMGB1‐mediated TLR4/NF‐κB and PI3K/AKT/mTOR pathways.^39^ Recent studies also indicated that PL can exert its anticancer pharmacological effects by regulating the PI3K/AKT/mTOR signalling.^36^ Zhang et al. indicated that PL treatment reduced the proliferation of human bladder cancer cells by suppressing the activation of PI3K/AKT/mTOR signalling.^40^ Our results suggested that the expression of p‐PI3K, p‐AKT and p‐mTOR in lung tissues were significantly increased during ALI, while PL does‐dependently inhibited the expression of these proteins induced by LPS in mice. In order to further verify the mechanism of PL anti‐ALI, we conducted experiments in vitro. The chosen concentration for cell experiments was 1–4 μM, as our experiments showed PL no effect on RAW 264.7 cell viability at this concentration. However, some studies suggested that PL with 2 μM can promote SMMC‐7721 cell apoptosis.^41^ Other studies suggested PL of 20 μM had no effect on the activity of MC3T3‐E1 cells.^42^ LPS stimulation of RAW264.7 cells resulted in the increase of p‐PI3K, p‐AKT and p‐mTOR expression and inflammatory cytokines TNF‐α, IL‐1β, and IL‐6 levels, while these changed were significantly reduced after treating with PL. However, the activation of PI3K/AKT/mTOR signalling by 740Y‐P significantly reversed the protective effect of PL on the LPS‐stimulated inflammatory response. Data in this study indicated that PL can ameliorates ALI induced by LPS by inhibiting the activation of the PI3K/AKT/mTOR signalling pathway.

Oxidative stress‐induced injury is a crucial contributor to lung failure, and the Keap1‐Nrf2/HO‐1 signalling participates in the regulating of oxidative stress. The regulated mechanism is associated with the antioxidant enzymes including CAT, SOD, GSH and Nrf2/HO‐1 signalling against oxidative stress.^18^ In normal circumstances, Nrf2 binds to Keap1 in the cytosol. After a redox imbalance occurs, Nrf2 dissociates from Keap and activates the expression of detoxification genes by binding with antioxidant response elements.^17^, ^18^ It has been reported that the Keap1‐Nrf2/HO‐1 signalling is inhibited during ALI,^43^ and the activation of Keap1‐Nrf2/HO‐1 signalling by panaxydol can significantly inhibit LPS‐induced ALI.^34^ In this study, we analysed the levels of GSH, SOD, CAT and MDA in lung tissues. The results showed that PL treatment reversed the changes in these enzymes induced by LPS. In addition, the expression of Keap1 was increased, and the expression of Nrf2 and HO‐1was decreased induced by LPS both in vivo and in vitro, while these changes were inhibited by PL treatment. Furthermore, inhibiting the activation of the Keap1‐Nrf2/HO‐1 pathway also hindered the protective effect of PL against inflammatory response induced by LPS. The results indicated that the activation of Keap1‐Nrf2/HO‐1 signalling is linked to the protective role of PL in the treatment of ALI.

## 5 CONCLUSION

The study discovered that PL effectively alleviated LPS‐induced ALI in mice by regulating the PI3K/AKT/mTOR and Keap1‐Nrf2/HO‐1 signalling. However, there are still many limitations in this study, as it did not use PI3K or mTOR or Keap1 or HO‐1 signal‐related gene knockout mice or cells to further verify whether PL affects ALI through altering the signalling pathway. Moreover, this study preliminarily demonstrated that PL has an inhibitory effect on LPS‐induced lung injury. However, it is unclear whether PL has the same effect on lung injury caused by bacterial infection. Additionally, the degradation time of PL in the lung requires further study. Further research will address these issues and provide a theoretical basis for the development of drugs to treat lung injury.

## AUTHOR CONTRIBUTIONS

**Zhengjia Liu:** Data curation (equal); investigation (equal); methodology (equal); writing – original draft (equal). **Jiahui Wei:** Investigation (equal); methodology (equal). **Hongbin Sun:** Methodology (equal); supervision (equal); validation (equal); writing – review and editing (equal). **Lei Xu:** Conceptualization (equal); funding acquisition (equal); project administration (equal); resources (equal); supervision (equal).

## FUNDING INFORMATION

This work was supported by the Education Department of Jilin Province (JJKH20211234KJ) and the Wu Jieping Medical Foundation (320.6750.19092–9).

## CONFLICT OF INTEREST STATEMENT

The authors have no financial interests to disclose.

## Data Availability

Data will be made available on request.
